# Supplementary material for: Preconditioning of sediment failure by astronomically paced weak-layer deposition
Source: Nat Commun. 2025 Aug 6;16:7244. doi: 10.1038/s41467-025-62493-4 (PMC12328812; doi:10.1038/s41467-025-62493-4)
Supplement: Supplementary file 1 — Supplementary Information [file 41467_2025_62493_MOESM1_ESM.pdf]

Supplementary Information

Preconditioning of sediment failure by astronomically paced weak-layer deposition

Xingxing Wang[1,2], Vittorio Maselli[3], Luca Flessati[4], Hongbin Wang[5], Zhilei Sun[5], Qing Wang[1], Jie Chen[6], Qing Li[5], Stefano Alberti[7], Markus Kienast[8], Shucheng Xie[9], Qiliang Sun[1,2,9,*]

1, Hubei Key Laboratory of Marine Geological Resources, China University of Geosciences, Wuhan 430074, China

2, Laboratory for Marine Mineral Resources, Qingdao Marine Science and Technology Center, Qingdao 266237, China

3, Department of Chemical and Geological Sciences, University of Modena and DATAReggio Emilia, Modena 41125, Modena, Italy

4, Faculty of Civil Engineering and Geosciences, Delft University of Technology, 2600 GA Delft, The Netherlands

5, Key Laboratory of Gas Hydrate, Ministry of Natural Resources, Qingdao Institute of Marine Geology, Qingdao 266237, China

6, School of Geosciences, China University of Petroleum (East China), Qingdao 266580, China

7, College of Forestry, Oregon State University, Corvallis OR 97331, United States

8, Department of Oceanography, Dalhousie University, Halifax, Nova Scotia B3H 4R2, Canada

9, State Key Laboratory of Geobiogeology and Environmental Changes, China University of Geosciences, Wuhan 430074, China

Corresponding authors:
sunqiliang@cug.edu.cn (Qiliang Sun)

1. Location of seismic profiles, ODP sites, and MTDs in the study area

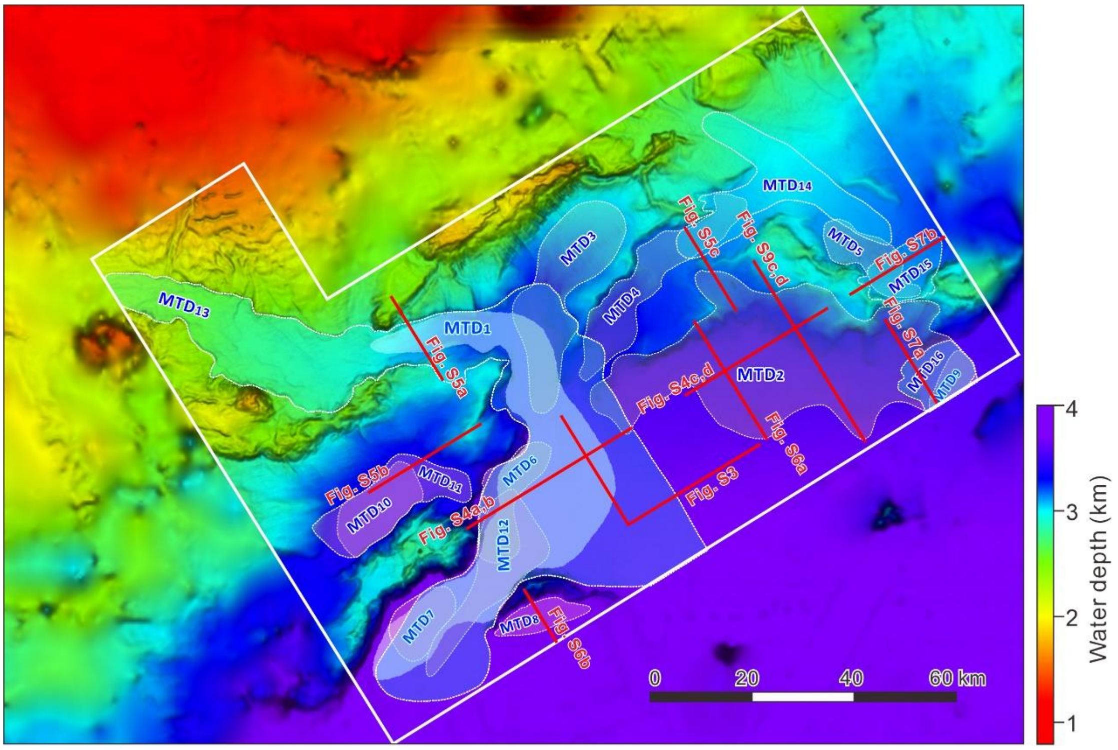

Figure S1. Location of seismic profiles and ODP sites 1145 and 1146 shown. Mass-transport deposits (MTDs) identified within the study area are highlighted. The bathymetry map outside the white boundary is based on the *GEBCO_2024 Grid* data[1].

2. Seismic facies description and interpretation

Seismic facies (SF) 1, characterized by sub-parallel, low- to medium-amplitude reflections, is the most diffuse. It is interpreted to represent hemipelagite deposits, often interbedded with fine-grained turbidites. In IODP Site 1499 and 1502 to the west of the study area, SF1 is characterized by the presence of abundant deep-water agglutinated benthic foraminifera, typical of bathyal environments[2,3], which support this interpretation. SF2, which is often laterally associated to SF 1 and primarily occurs in Quaternary sediments, shows high- to low-amplitude, wave-shaped to mounded reflections. SF2 is interpreted to represent sediment waves and contourite deposits, in agreement with other studies that identified the active role of ocean bottom currents in the SCS region during the Quaternary[4,5]. SF3, found in the deepest (pre-Quaternary) stratigraphic intervals imaged by the seismic profiles, is characterised by high-amplitude reflections, continuous to discontinuous, often intercalated with transparent units. SF3 is interpreted as coarse-grained turbidites and submarine fans[6,7]. SF4 consists of transparent to chaotic seismic reflections, often interbedded with contorted reflections, and are interpreted to represent mass-transport deposits (MTDs)[8–11] (Fig. S2).

Based on the recognition criteria of the MTDs on the seismic profiles and the regionally interpreted seismic surfaces, this study identified the top and bottom surfaces of the MTDs (Figs. S3-S7), and then obtained morphological parameters of the MTDs (Table S2).

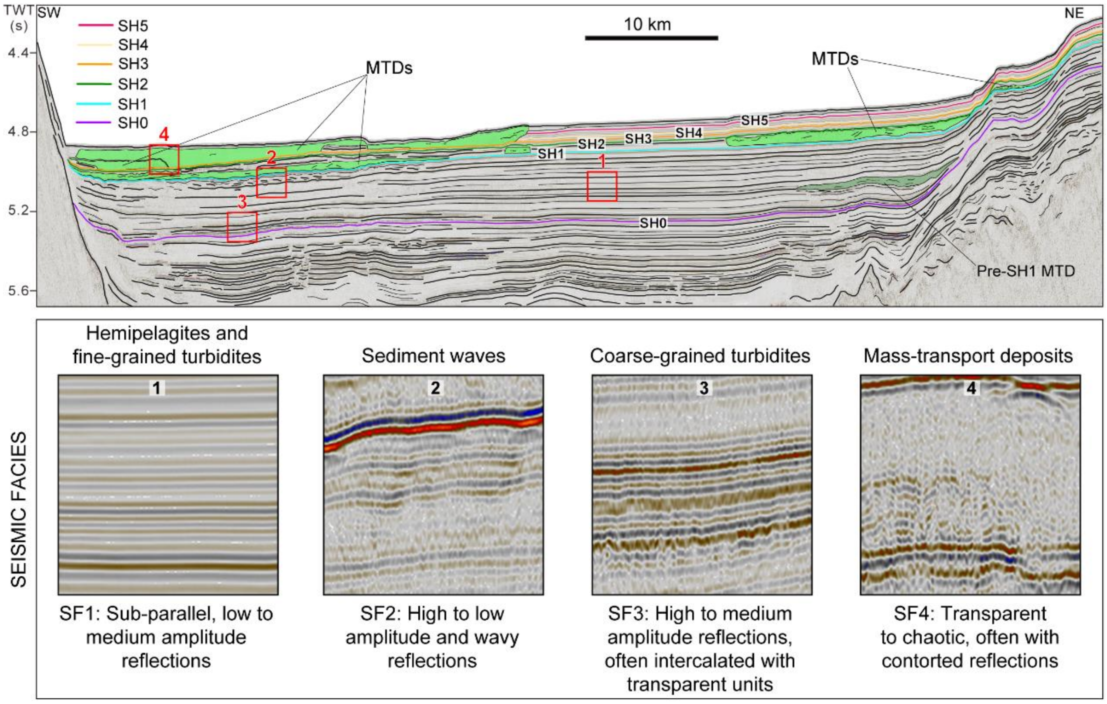

Figure S2. Main seismic facies identified in the study area. See location in Figs. 1 and 3 of the main manuscript.

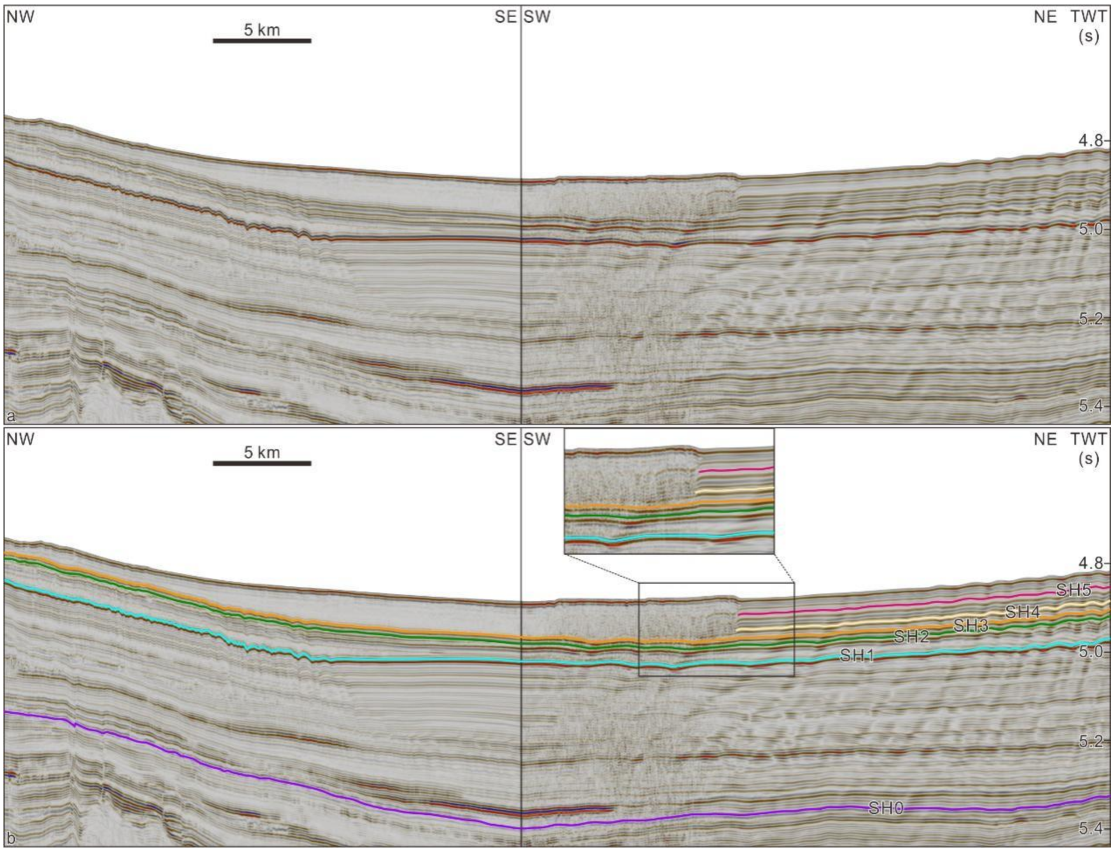

Figure S3. **(a)** Un-interpreted and (b) interpreted seismic profile showing the major seismic horizons. See location in Fig. S1.

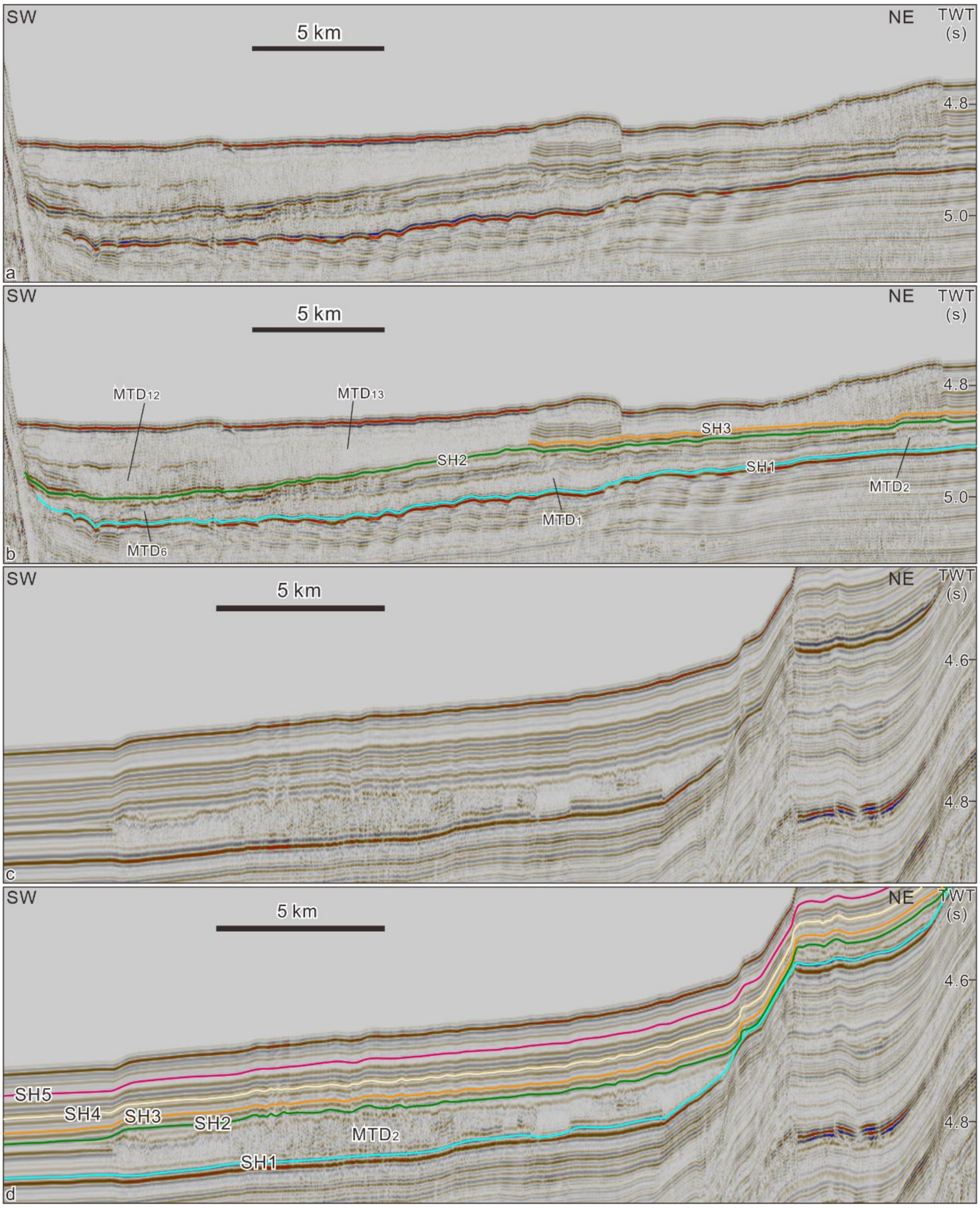

Figure S4. Close-up views of the seismic profile in Fig. 3 of the main manuscript, showing the different internal and external characteristics of the MTDs. Note the sharp interfaces between the MTDs and the adjacent undeformed strata.

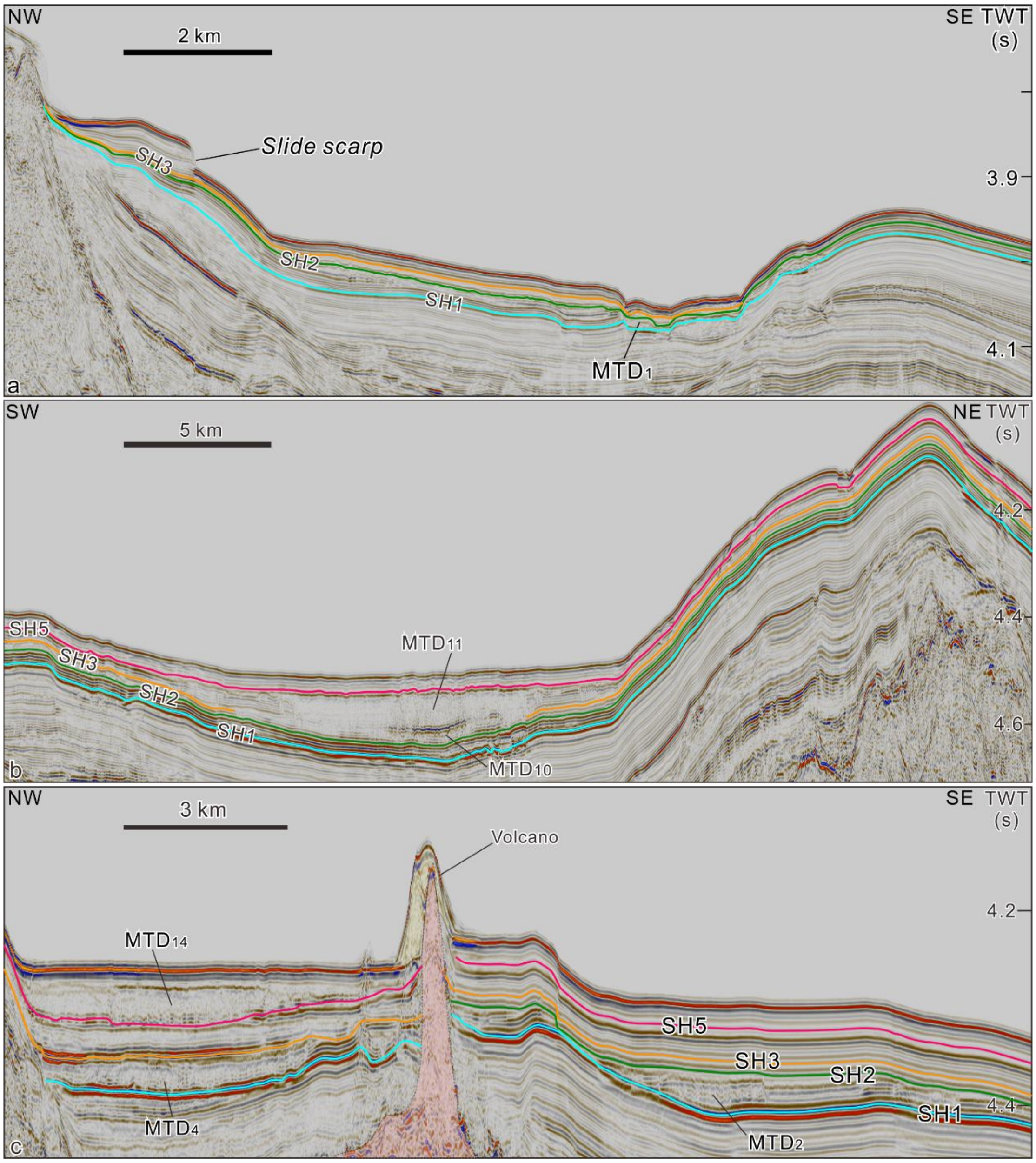

Figure S5. Seismic profiles showing the recurrent distribution of MTDs and their glide surfaces in the study area. The locations of the profiles in Fig. S1.

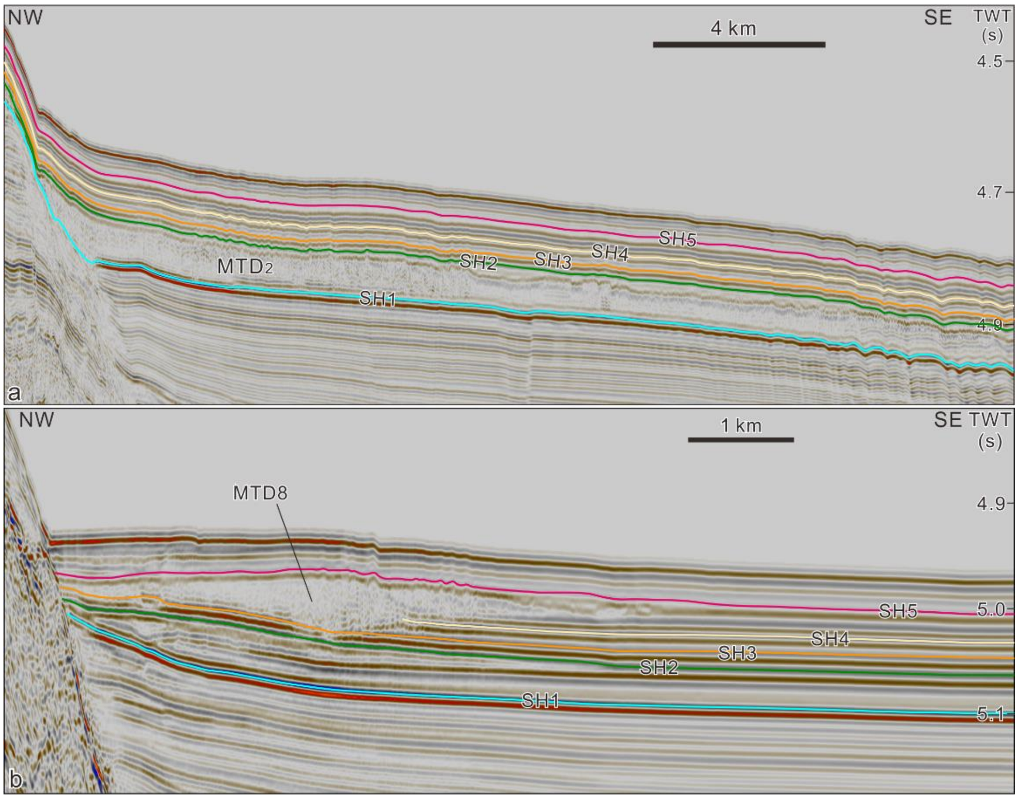

Figure S6. Seismic profiles showing the recurrent distribution of MTDs and their glide surfaces in the study area. The locations of the profiles in Fig. S1.

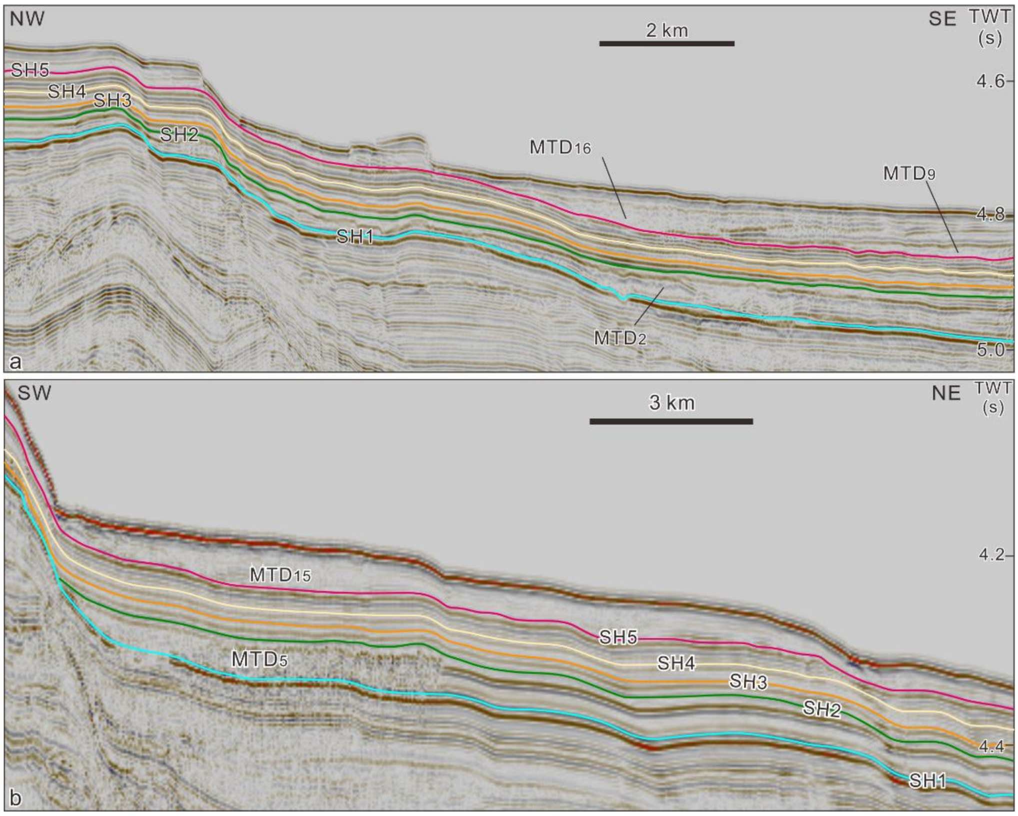

Figure S7. Seismic profiles showing the recurrent distribution of MTDs and their glide surfaces in the study area. The locations of the profiles in Fig. S1.

3. Morphological parameters of MTDs

Table S1. Morphological parameters for the MTDs identified in the study area.

MTDs	Basal surface/ weak layer	Coverage area (km^2)	Maximum thickness (ms)	Average Thickness (ms)
MTD1	SH1	1462	305	34
MTD2	SH1	1412	141	40
MTD3	SH1	401	92	34
MTD4	SH1	296	170	34
MTD5	SH1	127	80	42
MTD6	SH1	176	123	57
MTD7	SH3	120	57	32
MTD8	SH4	73	52	25
MTD9	SH5	73	44	26
MTD10	SH2	166	50	27
MTD11	SH4	275	100	32
MTD12	SH2 and SH3	275	60	34
MTD13	SH2 and SH3	2850	145	44
MTD14	SH4	418	60	32
MTD15	SH5	140	32	16
MTD16	SH5	130	44	21

4. Principal Component Analysis

Table S2. Element loadings of PC1 and PC2 (Fine- and coarse-end members are indicated by the sediments with grain-size modes of 4 μm and 19 μm, respectively).

Sedimentary parameters	Coefficients of PC1	Coefficients of PC2
Opal	0.424239869	-0.005830144
Porosity	0.387502016	-0.179816642
$\delta^{18}O$	0.326690499	0.169004951
Quartz	0.408342192	-0.179016873
Clay	-0.373326267	0.199903448
Fine end member	-0.183422438	-0.571190241
Coarse end member	0.181146146	0.603486052
Sedimentation Rate	0.373449361	0.112723407
Smectite/(Chlorite+Illite)	-0.226048776	0.404846402

5. Inversion of P-wave velocity at ODP Site 1145

The P-wave velocities (V_p) at ODP Site 1145 was calculated from the simplified three-phase equation (STPE)[12,13]. It is given by equation (1):

$$V_p = \sqrt{\frac{k + 4\mu/3}{\rho}} \quad (1)$$

where, the k, μ and ρ denote bulk modulus, shear modulus and density of the deposition medium, respectively. The bulk modulus k and the shear modulus μ of the deposition medium can be calculated by the following equation (2) and (3):

$$k = K_{ma}(1 - \beta_p) + {\beta_p}^2 K_{av} \quad (2)$$

$$\mu = \mu_{ma}(1 - \beta_s) \quad (3)$$

where, the K_{ma} and μ_{ma} denote the bulk modulus and shear modulus of rock skeleton, respectively. The K_{av}, β_p, and β_s can be calculated by the following equation (4):

$$\frac{1}{K_{av}} = \frac{\beta_p - \varphi}{K_{ma}} + \frac{\varphi}{K_w}, \ \beta_p = \frac{\varphi(1+\alpha)}{1+\alpha\varphi}, \ \beta_s = \frac{\varphi(1+\gamma\alpha)}{1+\gamma\alpha\varphi} \quad (4)$$

where, the K_W and φ denote the bulk modulus of water and the porosity of sedimentary medium, respectively. The parameters related to shear modulus γ and consolidation parameter α is calculated using equation (5) and (6):

$$\gamma = (1 + 2\alpha)/(1 + \alpha) \quad (5)$$

$$\alpha_i = \alpha_0 (P_0/P_i)^n \approx \alpha_0 (d_0/d_i)^n \quad (6)$$

where, α_0 is consolidation parameter at effective pressure p_0 and depth d_0, while α_i

is the consolidation parameter at effective pressure p_i and depth d_i. The consolidation parameters depend on the degree of consolidation and the effective pressure in the area. Mindlin (1949) suggests that the bulk modulus and shear modulus are 1/3 power of the effective pressure[14]. The consolidation parameter $\alpha_i = 42(60/d_i)^{1/3}$ was used in this study. Where α is the consolidation parameter and d is depth below sea floor in meter.

The density of deposition medium in equation (1) can be calculated by the following equation (7):

$$\rho = \rho_s(1-\varphi) + \rho_w\varphi \quad (7)$$

where, the ρ_s and ρ_w denote the density of rock skeleton and the density of pore water, respectively.

The bulk modulus K_{ma} and shear modulus μ_{ma} of rock skeleton in equation (3) are calculated by Hill's average equation[15] (8):

$$k_{ma} = \frac{1}{2}[\sum_{i=1}^{m} f_i k_i + (\sum_{i=1}^{m} f_i / k_i)^{-1}], \quad \mu_{ma} = \frac{1}{2}[\sum_{i=1}^{m} f_i \mu_i + (\sum_{i=1}^{m} f_i / \mu_i)^{-1}] \quad (8)$$

where, the m is the number of minerals in the solid part of the rock. The f_i denote the volume fraction of the ith mineral in the solid phase. The k_i is the bulk modulus of the ith mineral. The μ_i is the shear modulus of the ith mineral.

The density data, the porosity from ODP Site 1145 and the empirical elastic constants (Table S3) were used for the simplified three-phase equation (STPE).

Table S3 Constants used for the calculation of P-wave velocity

Bulk modulus (GPa)	Shear modulus (GPa)	Density(Kg/m^3)
$K_c = 20.9$	$G_c = 6.6$	$\rho_c = 2580$
$K_q = 38$	$G_q = 44$	$\rho_q = 2650$
$K_{cal} = 76.8$	$G_{cal} = 32$	$\rho_{cal} = 2710$
$K_w = 2.29$	$G_w = 0$	$\rho_w = 1000$

Annotate: subscripts *c*, *q*, *cal*, and *w* are for clay, quartz, calcite, and water, respectively. *K*, *G*, and *ρ* are bulk modulus, shear modulus, and density, respectively. The properties are from the refs. 12,13,16-18.

6. Composition of opal in ODP Site 1146.

The ref. 19 presented a detailed investigation of the opal accumulating on the northern South China Sea margin, based on data from ODP Site 1146. Their results show that the opal is mainly composed of radiolarians and diatoms, and much lower amounts of sponges (Fig. S8).

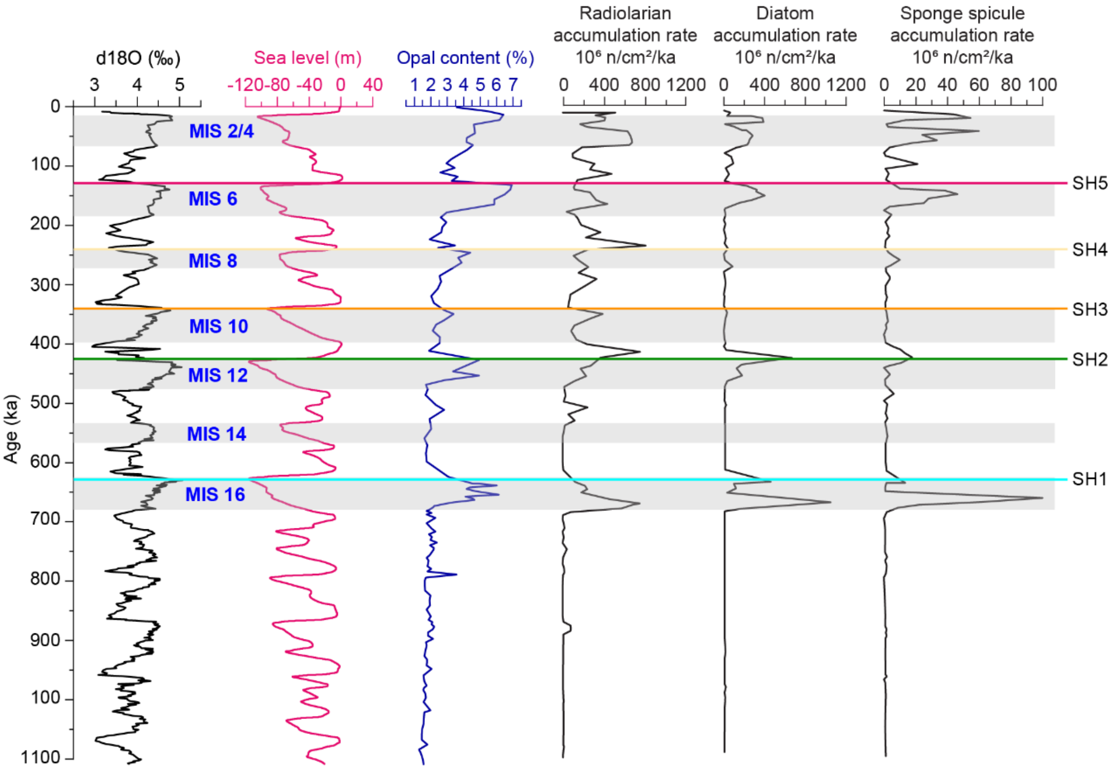

Figure S8. Correlation of the oxygen isotope record with sea-level fluctuations and accumulation rates of radiolarian, diatom and sponge at ODP Site 1146 (Modified from ref. 19).

7. Seismic-well tie of Site 1145

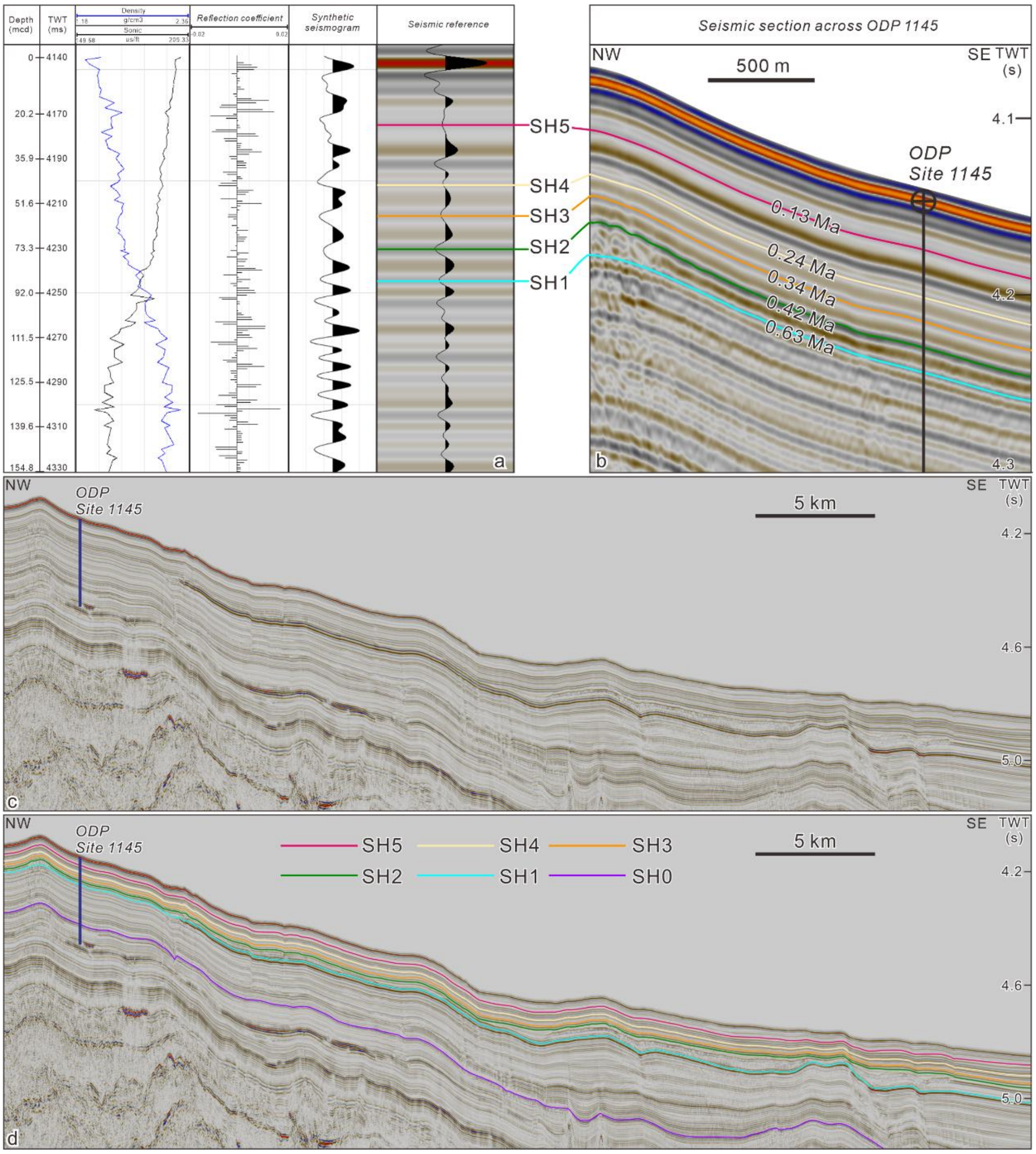

Figure S9. (a) Seismic-to-well tie of the ODP Site 1145; (b) Close-up view of the seismic profile with identified seismic horizons at ODP Site 1145; (c) Un-interpreted and (d) interpreted seismic profile across ODP Site 1145. Location in Fig. S1.

8. Plastic and liquid limit

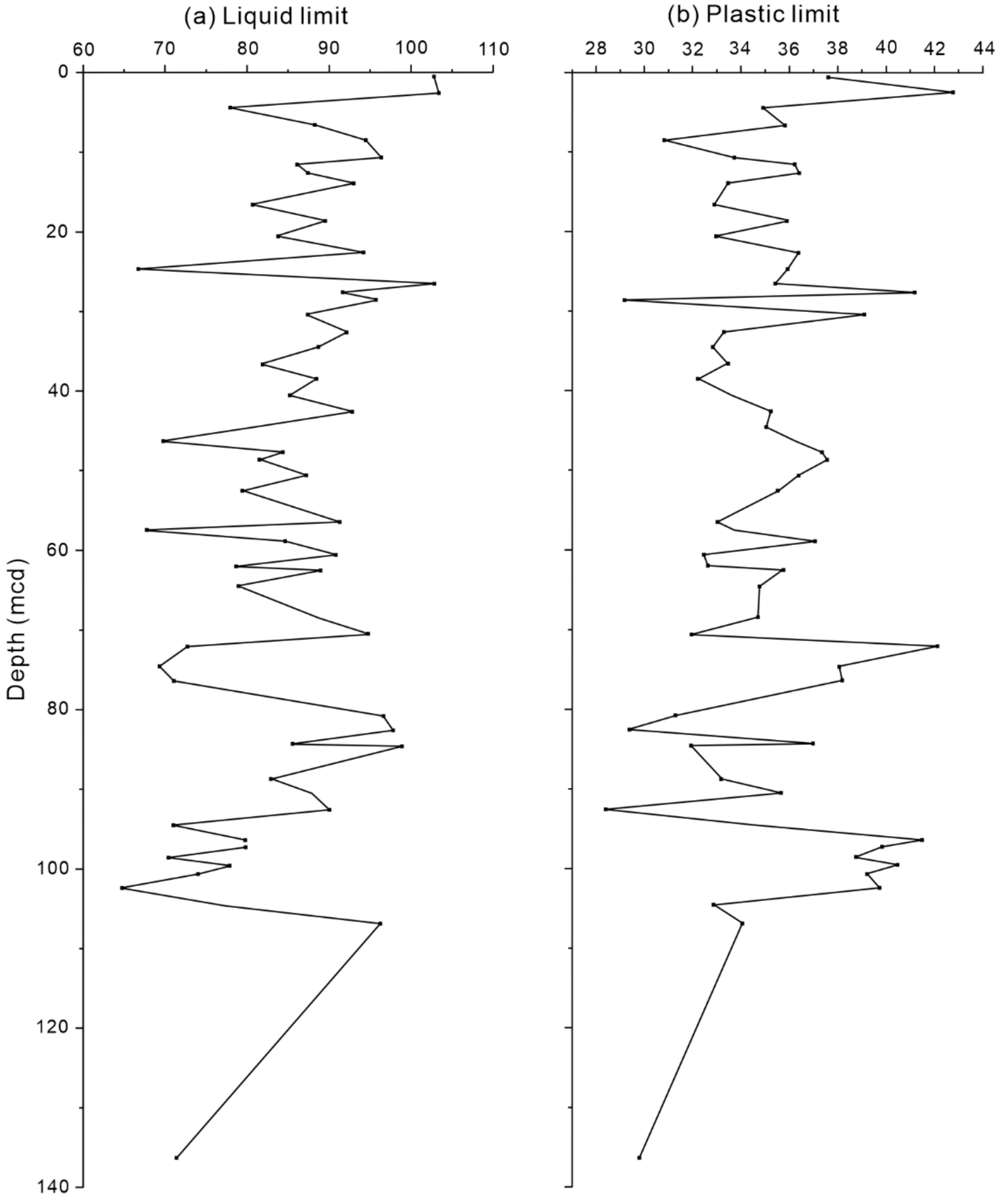

Figure S10. Liquid limit (a) and plastic limit (b) variation with depth for the ODP Site 1146 (the depth of each sample refers to the middle depth of the 10 cm-long section where the sample was collected to test)

References:

1. GEBCO Compilation Group. GEBCO 2024 Grid. https://doi.org/10.5285/1c44ce99-0a0d-5f4f-e063-7086abc0ea0f (2024).
2. Sun, Z. et al. Proceedings of the International Ocean Discovery Program Volume 367/368. (2018).
3. Jian, Z. et al. Discovery of the marine Eocene in the northern South China Sea. National Science Review 6, 881–885 (2019).
4. Wang, X. et al. Late miocene–quaternary seismic stratigraphic responses to tectonic and climatic changes at the northeastern margin of the south China sea. GSA Bull. 134, 2611–2632 (2022).

5. Yin, S. et al. Plate convergence controls long-term full-depth circulation of the South China Sea. Marine Geology 459, 107050 (2023).
6. Chen, H. et al. Depositional architecture and evolution of basin-floor fan systems since the Late Miocene in the Northwest Sub-Basin, South China Sea. Marine and Petroleum Geology 126, 104803 (2021).
7. Mayall, M. & Kneller, B. Seismic interpretation workflows for deep-water systems: A practical guide for the subsurface. Bulletin 105, 2127–2157 (2021).
8. Haflidason, H. et al. The Storegga Slide: architecture, geometry and slide development. Marine Geology 213, 201–234 (2004).
9. Moscardelli, L., Wood, L. & Mann, P. Mass-transport complexes and associated processes in the offshore area of Trinidad and Venezuela. Bulletin 90, 1059–1088 (2006).
10. Bull, S., Cartwright, J. & Huuse, M. A review of kinematic indicators from mass-transport complexes using 3D seismic data. Marine and Petroleum Geology 26, 1132–1151 (2009).
11. Alves, T. M. Submarine slide blocks and associated soft-sediment deformation in deep-water basins: A review. Marine and Petroleum Geology 67, 262–285 (2015).
12. Lee, M. W. Models for Gas Hydrate-Bearing Sediments Inferred from Hydraulic Permeability and Elastic Velocities. (2008).
13. Lee, M. W. & Collett, T. S. Scale-dependent gas hydrate saturation estimates in sand reservoirs in the Ulleung Basin, East Sea of Korea. Marine and Petroleum Geology 47, 195–203 (2013).
14. Mindlin, R. D. Compliance of Elastic Bodies in Contact. Journal of Applied Mechanics 16, 259–268 (1949).
15. Hill, R. S. R. The Elastic Behaviour of a Crystalline Aggregate. Proceedings of the Physical Society. Section A (1952) doi:10.1088/0370-1298/65/5/307.
16. Mavko, G., Mukerji, T. & Dvorkin, J. The Rock Physics Handbook: Tools for Seismic Analysis of Porous Media (Second Edition). (Cambridge University Press, Cambridge, 2009).
17. Waite, W. F., Kneafsey, T. J., Winters, W. J. & Mason, D. H. Physical property changes in hydrate-bearing sediment due to depressurization and subsequent repressurization. Journal of Geophysical Research: Solid Earth 113, (2008).
18. Waite, W. F. et al. Physical properties of hydrate-bearing sediments. Reviews of Geophysics 47, (2009).
19. Wang, R., Clemens, S., Huang, B. & Chen, M. Quaternary palaeoceanographic changes in the northern South China Sea (ODP Site 1146): radiolarian evidence. J. Quaternary Sci. 18, 745–756 (2003).
