# Supplementary material for: Preconditioning of sediment failure by astronomically paced weak-layer deposition
Source: Nat Commun. 2025 Aug 6;16:7244. doi: 10.1038/s41467-025-62493-4 (PMC12328812; doi:10.1038/s41467-025-62493-4)
Supplement: Supplementary file 2 — Description of Additional Supplementary Files [file 41467_2025_62493_MOESM2_ESM.pdf]

Description of Additional Supplementary Files

File name: Supplementary Data 1
Description: The Atterberg limit results and the calculated ratio of su/σ'v.

File name: Supplementary Data 2
Description: The original data of the Atterberg limit tests.
